# Dried Saliva Spots: A Robust Method for Detecting *Streptococcus pneumoniae* Carriage by PCR

**DOI:** 10.3390/ijms17030343

**Published:** 2016-03-05

**Authors:** Cassandra L. Krone, Anna E. Oja, Kirsten van de Groep, Elisabeth A. M. Sanders, Debby Bogaert, Krzysztof Trzciński

**Affiliations:** Pediatric Immunology and Infectious Diseases, Wilhelmina Children’s Hospital, University Medical Center Utrecht, 3508 AB Utrecht, The Netherlands; C.L.Krone@bham.ac.uk (C.L.K.); a.oja@sanquin.nl (A.E.O.); K.vandegroep-3@umcutrecht.nl (K.G.); L.Sanders@umcutrecht.nl (E.A.M.S.); D.Bogaert@umcutrecht.nl (D.B.)

**Keywords:** pneumococcus, colonization, surveillance, saliva, dried spots, upper respiratory tract

## Abstract

The earliest studies in the late 19th century on *Streptococcus pneumoniae* (*S. pneumoniae*) carriage used saliva as the primary specimen. However, interest in saliva declined after the sensitive mouse inoculation method was replaced by conventional culture, which made isolation of pneumococci from the highly polymicrobial oral cavity virtually impossible. Here, we tested the feasibility of using dried saliva spots (DSS) for studies on pneumococcal carriage. Saliva samples from children and pneumococcus-spiked saliva samples from healthy adults were applied to paper, dried, and stored, with and without desiccant, at temperatures ranging from −20 to 37 °C for up to 35 days. DNA extracted from DSS was tested with quantitative-PCR (qPCR) specifically for *S. pneumoniae*. When processed immediately after drying, the quantity of pneumococcal DNA detected in spiked DSS from adults matched the levels in freshly spiked raw saliva. Furthermore, pneumococcal DNA was stable in DSS stored with desiccant for up to one month over a broad range of temperatures. There were no differences in the results when spiking saliva with varied pneumococcal strains. The collection of saliva can be a particularly useful in surveillance studies conducted in remote settings, as it does not require trained personnel, and DSS are resilient to various transportation conditions.

## 1. Introduction

*Streptococcus pneumoniae* (*S. pneumoniae*) is an inhabitant of the human respiratory tract and frequently carried asymptomatically. On rare occasions, it breaches the host’s immune barrier and becomes a pathogen causing a range of diseases; including otitis media, pneumonia, bacteremia, and meningitis [1,2]. In general, pneumococcal colonization is considered a necessary precursor to disease [3], with a direct link between strains circulating in carriage and disease [4,5]. This link is commonly utilized by epidemiological surveillance studies targeting asymptomatic colonization in order to assess the effects of therapeutic and preventive strategies [6,7,8].

For the past several decades, the nasopharynx has been considered the optimal sampling niche for detection of *S. pneumoniae* colonization [6,7,8,9]. Yet a look into historical research reveals that saliva was the preferred diagnostic specimen [10]. Both Pasteur and Sternberg independently discovered *S. pneumoniae* in 1881 by infecting animals with human saliva, and, for more than half a century, testing saliva in mice was considered the optimal method for carriage detection [10,11]. Cross-sectional studies using this method in the pre-antibiotic era identified 45% to 60% of adults [10,11] and 50% to 80% of schoolchildren as asymptomatic carriers [12]. With the rise of antibiotic use, and the progress in culture-based diagnostic methods, interest in the use of saliva for epidemiological surveillance declined. Furthermore, the sensitivity of conventional cultures was low when used for recovering live pneumococci from highly polymicrobial saliva samples [10], contributing further to the downfall of oral fluids as diagnostic specimens in pneumococcal studies. However, recent progress in culture-independent molecular methods has led to a dramatic increase in sensitivity and specificity of pathogen detection [9,13,14,15,16], and prompted our interest in re-visiting saliva as a specimen for epidemiological studies on *S. pneumoniae* carriage.

Saliva is an easily accessible, easy-to-collect body fluid secreted into the oral cavity at the crossover of the digestive and respiratory tracts. The general understanding is that preserving saliva samples requires storage and transport at low temperatures, preferably snap frozen and transported on dry ice [17,18]. The requirement for a cold chain would therefore complicate the use of saliva as a diagnostic specimen for pneumococcal detection. However, historical studies provide alternative solutions for the storage and preservation of saliva samples; Nissen was the first to report on drying pneumococci for later use in 1891 [19]. By the 1930s dehydration was considered “an excellent physical method for the preservation of cultures of Pneumococcus, particularly… (for) long period(s) of time (that) require that the characters of the strain be held uniform and constant” [11].

The dried spot method is widely used to collect and preserve blood and other body fluids for a range of diagnostic purposes [20,21,22,23,24]. We therefore evaluated dried saliva spots stored at various temperatures as a method for detecting pneumococcal presence in saliva of asymptomatic individuals. The presence of pneumococci was detected using quantitative PCR (qPCR).

## 2. Results

### 2.1. Optimization of Dried Saliva Spots

**Figure 1:**
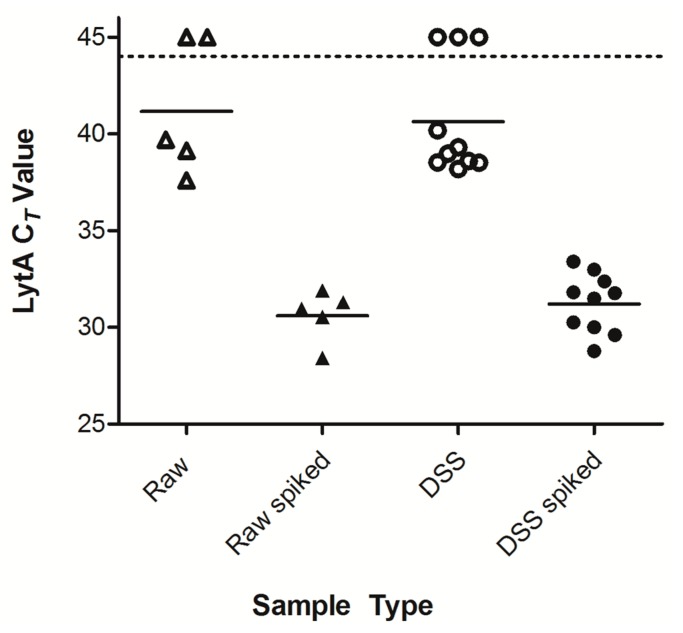
*Streptococcus pneumoniae* (*S. pneumoniae*) detection in raw saliva *versus* dried saliva spots (DSS). Saliva samples from 5 donors were spiked with a serotype 19F strain of *S. pneumoniae.* Both spiked and un-spiked raw saliva and DSS specimens were processed for DNA isolation and quantitative-PCR (qPCR)-based pathogen detection. Each dot represents an individual sample (DSS were performed in duplicate). The dashed line marks the lower limit of detection of qPCR. Differences between un-spiked raw and un-spiked DSS specimens as well as spiked raw and spiked DSS specimens samples are not significant.

To examine the efficiency of recovery of *S. pneumoniae*-specific DNA from saliva samples collected with dried saliva spots (DSS), we compared raw saliva to DSS. Individual samples from five donors were used to examine the effect that drying saliva on filter paper had on the recovery of pneumococcal DNA. A comparison of DSS and fresh samples spiked with live cells of serotype 19F *ATCC6319* strain of *S. pneumoniae* revealed no significant difference in quantity of the *S. pneumoniae-*specific gene *lytA* detected by qPCR (*p* = 0.450), as was the case for un-spiked samples (*p* = 0.753) (Figure 1). The detection of *lytA* in un-spiked donor saliva was not altogether surprising, as our previous studies show penumococccal colonization is present in the general population of adults in the Netherlands [13,16]. Additionally, testing un-spiked saliva allowed us to determine the baseline *lytA* signal before the sample was spiked.

We studied the effect of humidity on sample quality, since it had been reported that storing dried blood spots with desiccant improves DNA stability over time [25,26]. Indeed we found a decrease in *S. pneumoniae*-specific signal of the value 2 to 3 cycle threshold (C*_T_*) (increase in C*_T_* corresponds to a decline in signal strength) at day 7 in samples stored without desiccant. With the clear benefit of lowering humidity levels for DSS storage, we continued all further experiments with desiccant. We also tested the survival of *S. pneumoniae* in DSS and found that no live pneumococci recovered from freshly dried DSS, with few oral commensals surviving (~10 colony forming unit per spot (CFU/spot). This killing effect was equally present when spots were inoculated with cells suspended in phosphate buffered saline (PBS), but not when desiccated on plastic Petri dishes, indicating a strong bactericidal effect of the filter paper itself.

Considering that DSS is an unexplored method for pneumococcal detection, we assessed the lower limit of *S. pneumoniae* detection in DSS. We first tested the sensitivity of the molecular method by quantifying *lytA* presence in DNA extracted from a serial dilution of bacteria in PBS. In the absence of saliva components that could potentially interfere with pathogen detection, the qPCR method itself showed reproducible quantification in samples containing the equivalent of approximately 10 CFU per qPCR reaction with the lower limit of detection (LOD) of 6 CFU/reaction. Then, we tested the DSS method, spiking the saliva with serially diluted pneumococci. The results for DSS were also highly reproducible and precise for samples with ≥10^2^ CFU per qPCR reaction (LOD = 25 CFU/reaction), equivalent to approximately 10^4^ CFU per spot.

### 2.2. Temperature Stability

**Figure 2:**
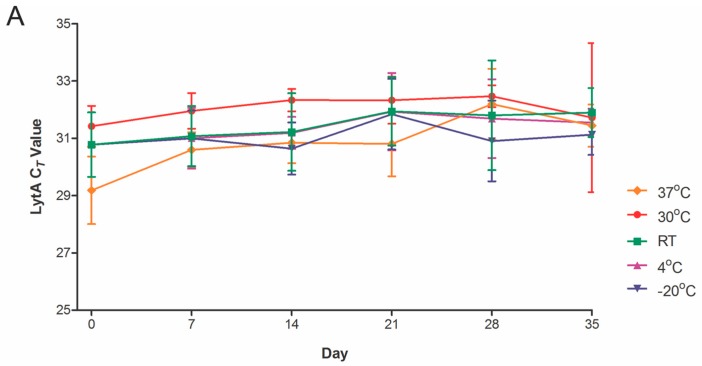

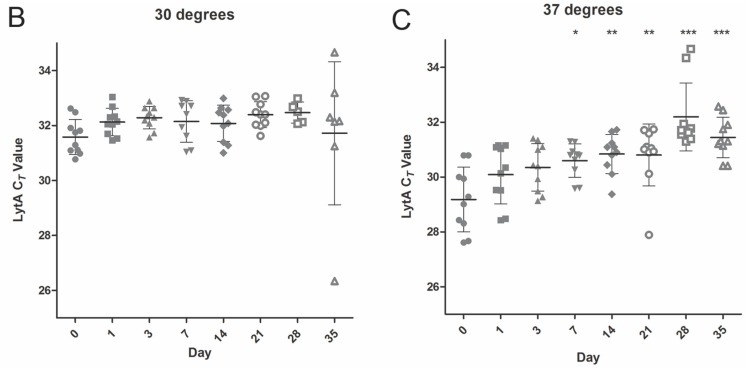
Pneumococcal signal in DSS is stable over long periods of time. Mock DSS specimens from saliva (*n* = 5 donors per experiment) spiked with *S. pneumoniae* serotype 19F. (**A**) DNA was isolated immediately after drying (day 0) and after storing the DSS at the indicated temperatures for up to 35 days. Shown are the mean and SD of all cycle threshold (C*_T_*) values. For 30 °C, room temperature, 4 or −20 °C, no significant differences were found for C*_T_* values of DSS stored over time compared to C*_T_* values at day zero; (**B**) Graph of individual DSS stored at 30 °C; (**C**) Graph of individual DSS stored at 37 °C for up to 35 days: a gradual loss of signal was observed at 37 °C but not at ≤30 °C (one-way ANOVA with Bonferroni post-test * *p* < 0.05, ** *p* < 0.001, *** *p* < 0.0001).

DSS specimens generated from saliva of five individual donors spiked with pneumococcal cells were stored for up to five weeks at five different temperature conditions, ranging from −20 to 37 °C, and tested weekly for pneumococcal-specific DNA by qPCR. Whenever possible, duplicates of DSS were tested throughout the course of time. Although we observed variation in the signal strength over time, there was no evidence of a significant decline of pneumococcal-specific signals in DSS specimens stored for up to one month at temperatures ≤30 °C (Figure 2A,B) or stored for up to 7 days at 37 °C (Figure 2C). The results were similar for DSS samples of saliva collected from various donors.

### 2.3. Short-Term DNA Stability of DSS of Different Strains of Streptococcus pneumoniae (S. pneumoniae)

**Figure 3:**
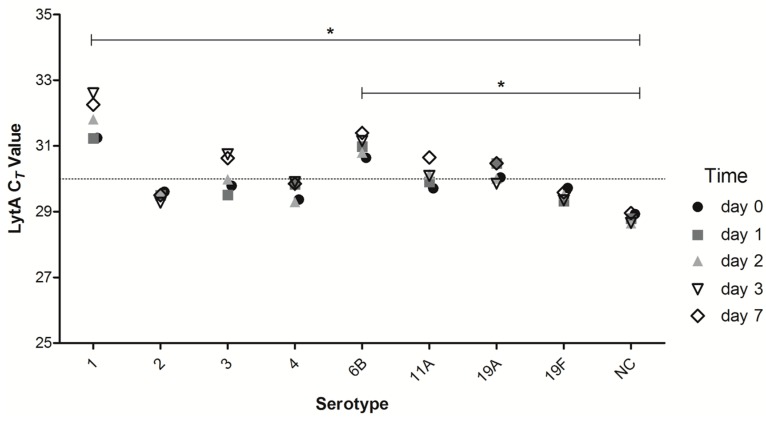
Effect of saliva on various strains. Saliva of 1 donor was aliquoted and spiked with different serotype strains, after which DSS were generated. DSS were analyzed for pneumococcal-specific signal at day 0, 1, 2, 3 and 7. Dots are the mean C*_T_* of 3 experiments (different donor each experiment). DSS specimens were kept at RT for the duration of the experiment. NC: non-encapsulated strain. Stable C*_T_* values are observed for each individual serotype/strain. Comparing all serotypes, only two are significantly different: serotype 1 compared to NC and serotype 6B compared to NC (two-way ANOVA, Bonferroni post-test * *p* < 0.05).

A previous study showed that *S. pneumoniae* tolerance to desiccation is not strain-specific, although some clinical strains were more robust than others [27]. We examined if the same was true for pneumococcal DNA in DSS. To study this, we tested an additional seven clinical isolates of serotypes 1, 2, 3, 4, 6B, 11A, 19A, and an acapsular strain constructed in the lab (Table 1). Aliquots of saliva samples collected from three donors were individually spiked with cells of pneumococcal strains, spotted onto filter paper, and stored. Overall, pneumococcal DNA was stable for up to 7 days in all strains tested (Figure 3). In concordance with the aforementioned results for serotype 19F, the qPCR C*_T_* values were robust and showed only minimal variation over time. However, we observed inter-strain variation in the quantity of *S. pneumoniae*-specific signal detected at time zero. When comparing all strains, results for serotypes 1 and 6B were significantly different compared to the acapsular strain with mean 2.3 C*_T_* and 1.7 C*_T_* difference of DSS compared to raw saliva, respectively (Two-way ANOVA with Bonferroni post-test, *p* < 0.05), despite matching number of cells used to spike samples, suggesting a variation in strains’ sensitivity to lysis in human saliva. Each sample was also run in the corresponding serotype-specific qPCRs and qPCR targeting another.

*S. pneumoniae*-specific sequence within the *pia* iselet [13]. These signals were equally robust as the signal for the *lytA* gene (data not shown), indicating that DSS will be suitable for molecular determination of the serotype composition of the sample as well.

### 2.4. Detection of Pneumococcal Presence in DSS Specimens from Clinical Saliva Samples

**Figure 4:**
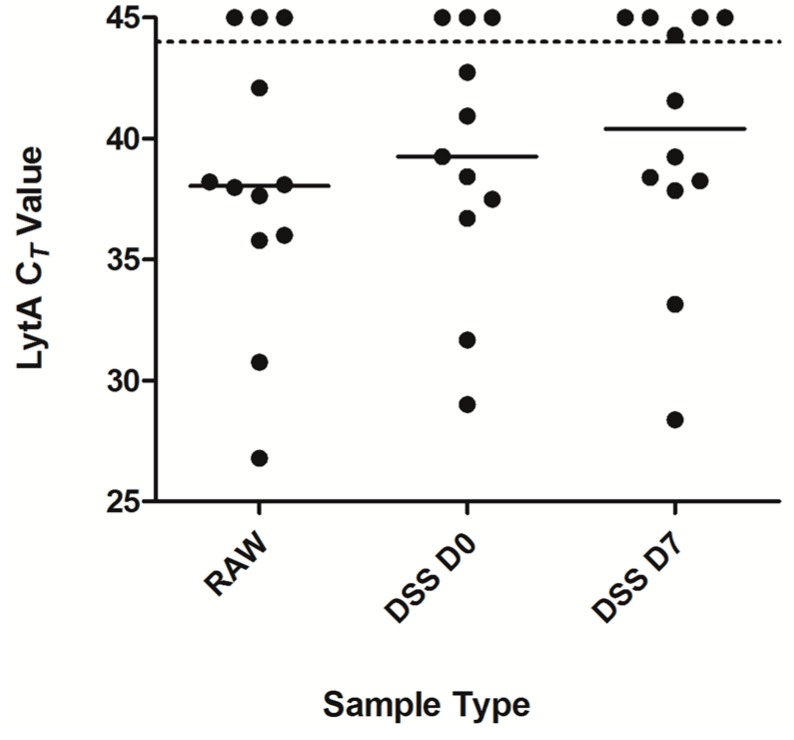
Detection of pneumococci in DSS of clinical samples. Saliva was collected from 12 school children (ages 4–10 years) and either frozen or processed for DSS immediately. DNA isolation was performed from raw saliva and DSS specimens from the same donors simultaneously. Samples are represented by dots with the median C*_T_* value depicted per condition. Pneumococcal-specific PCR identified 8 out of 12 children positive in both raw saliva and DSS at day zero. At 7 days at RT, 7 out of 12 children were positive for the pneumococcus-specific signal, indicating a loss of 1 positive result from sample weakly positive already at day zero.

In order to test the DSS method in a clinical setting, we tested saliva samples collected from 12 school children [15]. DNA was extracted from raw saliva and matching DSS, which were stored at RT for 7 days. When we used our strict criteria to assign positivity to a sample (presence of signals <45 C*_T_* for both *lytA* and *pia* in qPCR) [15], samples from 8/12 children were positive both in raw saliva and in DSS samples at day zero (Figure 4) and from 7/12 children at day 7.

In general, we observed a minimal decline in the signal detected in DSS compared to raw saliva samples (mean decline 0.88 C*_T_*, range 0.22–2.9 C*_T_*). Additionally, we tested the DSS and raw clinical samples for the presence of pneumococcal serotypes using serotype-specific qPCRs (data not shown). Differences in the C*_T_* values in serotype-specific qPCR for raw saliva compared to DSS were not significant.

## 3. Discussion

Historical records of sensitive mouse inoculation methods detecting high carriage rates in saliva of asymptomatic individuals triggered our interest in saliva as a diagnostic specimen to study pneumococcal carriage. It was further strengthened by the outcome of our recent study on pneumococcal carriage in the elderly [16], where we found saliva to be superior to nasopharyngeal and oropharyngeal sampling for *S. pneumoniae*. Additionally, two studies used dried spots for molecular detection of pneumococci in cerebral spinal fluid [24,34], and two recent studies have shown that dried saliva spots can be used to detect chemicals such as lidocaine [35] or lactic acid in saliva [36]. We hypothesized that the dried saliva spot (DSS) method could also be utilized for molecular detection of *S. pneumoniae* in highly polymicrobial saliva samples, simplifying sampling methodologies for studies on pneumococcal carriage. To our knowledge, this is the first attempt to explore the possibility of using DSS as a diagnostic tool for *S. pneumoniae* carriage detection.

We found that, despite processing through filter paper and desiccation, pneumococcal DNA was stable in DSS stored for up to one month and over a broad range of temperatures. This is in line with results reported by Peltola *et al.*, who detected pneumococcal DNA in cerebrospinal fluid after it had been applied to dried spots and stored at room temperature for up to 8 months [24]. The robustness of the DSS method provides the opportunity for unassisted sample collection. Saliva can be applied to DSS by individuals at home or in remote study centers, and sent via regular mail to the laboratorial facility for processing. This would be of particular advantage for surveillance studies on pneumococcal carriage conducted in resource-poor countries or remote areas.

Interestingly, at day zero, we already saw limited but significant inter-individual variation in the quantity of *S. pneumoniae*-specific signal detected in saliva samples spiked with an equal number of pneumococcal cells. Given that the C*_T_* value obtained for raw spiked saliva and for fresh or stored DSS was nearly constant for any one individual tested, it suggests that the variation is not the effect of drying, handling, or storing the saliva, but the intrinsic variability in saliva composition between individuals donors. This may be due to differences in composition of saliva itself (e.g., bactericidal molecules and enzymes produced by humans), the competition among microorganisms present in the oral cavity, and/or mediated by bacterial and fungal products and bacteriophages.

We also observed minor variation in quantity of *S. pneumoniae*-specific signal detected by qPCR in saliva from single donors spiked with different pneumococcal strains. This suggests the presence of inter-strain variation in sensitivity to bactericidal effects of saliva. We believe these limits can be easily addressed within the method. Since each qPCR reaction used only a fraction of the isolated DNA, increasing template volume, or concentrating the template, could further increase the sensitivity of the DSS method or any molecular-based method. Since saliva can be easily collected from a donor, sample volume should not be an important limiting factor.

The limitation of this study is the relative small sample size of saliva samples tested in the clinical part of the study. Another limitation is the inability to culture live pneumococci from the filter paper, which failed due to the apparent bactericidal effect of the paper used. Therefore, the DSS should be considered for studies not requiring isolation of live *S. pneumoniae* as the primary endpoint. However, the sterilizing effect of the DSS could also be considered beneficial, as it would reduce the biohazard associated with collecting and transporting the samples.

Further studies are needed to determine the diagnostic value of DSS compared to the gold standards, *i.e.*, conventional culture or molecular detection of pneumococci in nasopharyngeal swabs. Since this is the first description of this methodology, further validation on larger data sets, plus side-by-side comparisons with detection of pneumococcal carriage in other types of respiratory samples (nasal, nasopharyngeal or oropharynegeal swabs, nasal washes), will be necessary. Future research should aim to gain insight whether DSS has the potential to be used as a molecular quantitative method. For this, testing DSS samples from patients with pneumococcal disease or at risk of pneumococcal infection would be particularly informative. For use of this method in young or disabled individuals, we suggest using diagnostic kits designed for saliva collection before applying saliva on the filter paper. Further studies are needed to validate this additional step to the protocol.

In conclusion, DSS is an easy, novel, and robust saliva sampling method that shows promise as a tool for pneumococcal surveillances in remote areas and resource-poor countries. Pneumococcal DNA is stable in DSS stored with desiccant in a wide range of temperatures for up to one week. Furthermore, long-term storage of DSS is possible at −70 °C and consequently has great potential for diagnostic purposes. When molecular, culture-free methods are used for *S. pneumoniae* detection, DSS may be considered as an attractive alternative to nasopharyngeal or oropharyngeal swab samples in surveillance studies on pneumococcal carriage.

## 4. Materials and Methods

### 4.1. Bacterial Strains

The *S. pneumoniae* strains used in this study are listed in Table 1. Pneumococci were grown to mid-log phase in brain-heart infusion broth (BHI, Oxoid, Wesen, Germany), and aliquots were frozen in 10% glycerol at −80 °C. Prior to use, bacterial cells were thawed, washed twice with PBS, tittered by culturing tenfold dilutions on blood agar supplemented with gentamicin (SB7-Gent, Oxoid), and re-suspended in PBS to reach a particular CFU concentration. Experiments were performed with serotype 19F strain *ATCC6319*, unless specified otherwise.

### 4.2. Mock Saliva Experiments

Saliva samples were collected from nine healthy volunteer donors aged 21–49 years, 8 female and 1 male. Individuals were asked to expectorate saliva into a 50-mL polypropylene tube (Sarstedt, Nümbrecht, Germany), which was kept on ice. The saliva samples were considered whole mouth unstimulated saliva. Samples were vortexed vigorously for 10 s, and 100 µL of un-spiked saliva was stored for determination of baseline presence of *S. pneumoniae*. The remaining saliva was spiked with *S. pneumoniae* to a final concentration of approximately 10^6^ CFU/mL, and vortexed again.

### 4.3. Dried Saliva Spots

Immediately after spiking, 100 µL of saliva was used to inoculate the spot on a diagnostic filter paper card (Whatman 903 Protein Saver Card, VWR International, Amsterdam, The Netherlands) and dried at room temperature (RT) for 2 h; day zero samples were processed for *S. pneumoniae* detection when spots were fully dry. Unless specified otherwise, all DSS were stored in a sealed plastic zipper bag with a Minipax absorbent packet (Sigma-Aldrich, Zwindrecht, The Netherlands) as desiccant, and placed in darkness to limit light exposure. The DSS were processed in duplicate whenever possible. Samples were stored at the following temperatures: −20, 4, ~19 (RT), 30, and 37 °C.

### 4.4. Clinical Study

A small selection of samples were used from a study already conducted [15]. The study was conducted on a single day in June 2012, at a rural school of 190 students in the Utrecht province. Fifty students (aged 5 to 10 years, median 8 years) attending two different classes took part in the study. Except for the age of each student, no demographic data were collected. The study was conducted in line with the Ethical Committee Guidelines. Written informed consent was obtained from the parents of each child. Children were asked to spit saliva into a 15 mL polypropylene tube (Sarstedt), and 12 random samples were in parallel snap frozen and processed through DSS as described above. The saliva samples were considered whole mouth unstimulated saliva. One DSS sample at day zero was lost due to technical difficulties.

### 4.5. Isolation of Bacterial DNA

DNA was extracted with DNeasy Blood & Tissue Kit (Qiagen, Venlo, The Netherlands). DSS were cut out of the card and supplemented with 180 µL of 20 mM Tris-Cl, 2 mM EDTA; incubated for 15 min at 95 °C to inactivate DNAses; supplemented with equal volume (180 µL) of 2.4% Triton X-100, 80 mg/mL lysozyme in 20 mM Tris-Cl, 2 mM EDTA, vortexed and incubated at 37 °C for 30 min. The liquid phase was separated from the filter paper by pipetting, mixed with ethanol and processed according to the kit’s original protocol. DNA was eluted with 200 µL of an elution buffer and stored at 4 °C.

### 4.6. Real-Time Quantitative PCR Targeting S. pneumoniae 

Detection of *S. pneumonia*e-specific DNA was conducted by real-time qPCR using primers and probes specific for the gene coding for the major *S. pneumoniae* autolysin *lytA* [37] and for the *pia* iselet of the iron uptake ATP-binding cassette (ABC) transporter [13]. Genomic DNA of *S. pneumoniae* 19F was used as a positive control in qPCR, and, unless stated otherwise, 2.5 μL of DNA template was tested in a 25-μL PCR volume. It corresponded to approximately 1.25 × 10^3^ CFU of the artificially spiked saliva samples. Clinical samples were classified as positive for *S. pneumoniae* when C*_T_* values for both targeted genes were below 45 [38]. Serotype-specific genes were quantified using qPCR protocol published by Azzari *et al*. [14].

### 4.7. Lower Limit of S. pneumoniae Detection

We spiked saliva with a range of ten-fold PBS dilutions of pneumococcal cells from 10^5^ to 100 CFUs per 100-µL volume of saliva, and quantified *S. pneumoniae* presence in DNA extracted from DSS inoculated with these samples. Cell suspensions in PBS containing corresponding numbers of *S. pneumoniae* CFUs were processed as a reference curve.

### 4.8. Statistical Analysis

Results were analyzed using GraphPad Prism version 5.0 for Windows (GraphPad Software, San Diego, CA, USA). Due to small sample sizes, we assumed the data was normally distributed and used a Student’s *t*-test. For multiple comparisons, either one- or two-way ANOVA with Bonferroni post-tests were used as indicated.

## Figures and Tables

**Table 1:** *Streptococcus pneumoniae* strains used in this study.

Strain	Serotype	Description	Reference or Source
GA07694	1	Invasive clinical isolate	[28]
GA03901	2	Invasive clinical isolate	[29]
GA07650	3	Invasive clinical isolate	[29]
TIGR4	4	Invasive clinical isolate	[30]
603	6B	Invasive clinical isolate	[31]
603J	NT ^1^	Acapsular variant of 603	[28]
1101	11A	Invasive clinical isolate	[32]
SJD86	19A	Invasive clinical isolate	[33]
*ATCC6319*	19F	-	-

^1^ NT—non-typeable.

## Abbreviations

DSS	dried saliva spot
qPCR	quantitative polymerase chain reaction
DNA	deoxyribonucleaic acid
CFU	colony forming unit
C*_T_*	cycle threshold
LOD	limit of detection
